# The anticancer effect of the HDAC inhibitor belinostat is enhanced by inhibitors of Bcl‐x_L_ or Mcl‐1 in ovarian cancer

**DOI:** 10.1002/1878-0261.70050

**Published:** 2025-06-08

**Authors:** Cécilia Thomine, Sterenn Guillemot, Louis‐Bastien Weiswald, Romane Florent, Edwige Abeilard, Florence Giffard, Emilie Brotin, Mélanie Briand, Enora Dolivet, Laurent Poulain, Marie Villedieu

**Affiliations:** ^1^ Université de Caen Normandie, Inserm, Anticipe U1086, Interdisciplinary Research Unit for the Prevention and Treatment of Cancer, BioTICLA Laboratory (Precision Medicine for Ovarian Cancers) Caen France; ^2^ UNICANCER, Comprehensive Cancer Center François Baclesse Caen France; ^3^ Université de Caen Normandie, Services Unit PLATON, ORGAPRED Core Facility Caen France; ^4^ Université de Caen Normandie, Services Unit PLATON, VIRTUAL'HIS Core Facility Caen France; ^5^ Université de Caen Normandie, Services Unit PLATON, ImpedanCELL Core Facility Caen France; ^6^ Université de Caen Normandie, Services Unit PLATON, Biological Resources Center “OvaRessources” Caen France; ^7^ UNICANCER, Comprehensive Cancer Center François Baclesse, Biological Resources Center “OvaRessources” Caen France; ^8^ UNICANCER, Comprehensive Cancer Center François Baclesse, Department of Surgery Caen France

**Keywords:** Bcl‐x_L_ inhibitor, HDAC inhibitor, Mcl‐1 inhibitor, ovarian cancer, targeted therapy

## Abstract

Identifying innovative therapeutic strategies is crucial to improve clinical management of ovarian cancer. Previously, we showed that ovarian cancer cell apoptosis can be triggered by inhibiting the anti‐apoptotic proteins Bcl‐x_L_ and Mcl‐1 and/or by inducing their pro‐apoptotic partners Bim, Puma, and Noxa. The expression of these pro‐apoptotic proteins can be hindered by excessive histone deacetylation, resulting from HDAC overexpression. This study aimed to evaluate whether belinostat, an FDA‐approved pan‐HDAC inhibitor, could increase Bim, Puma, and/or Noxa expression and induce ovarian cancer cell death, either alone or in combination with strategies targeting Bcl‐x_L_ or Mcl‐1. Belinostat exerted a cytostatic effect and, at higher concentrations, an apoptotic effect in SKOV3 and IGROV1‐R10 ovarian cancer cells. It induced a concentration‐dependent increase in Bim, Puma, and Noxa protein expression, while partially repressing that of Bcl‐x_L_. Inhibition of Bcl‐x_L_ sensitized both cell lines to belinostat, as did inhibition of Mcl‐1 in IGROV1‐R10 cells. Interestingly, belinostat's anticancer activity was also enhanced by inhibitors of Bcl‐x_L_ or Mcl‐1 in patient‐derived tumor organoids. This study therefore positions belinostat‐based strategies as promising therapies for ovarian cancer.

## Graphical abstract

The pan‐HDAC inhibitor belinostat increases the expression of the pro‐apoptotic proteins Bim, Puma, and Noxa and induces apoptosis in ovarian cancer cell lines and patient‐derived tumor organoids when used at high concentrations. Moreover, inhibiting the anti‐apoptotic proteins Bcl‐x_L_ or Mcl‐1 sensitizes these preclinical models to the cytotoxic effect of belinostat. This study therefore positions belinostat‐based strategies as promising therapies for ovarian cancer.

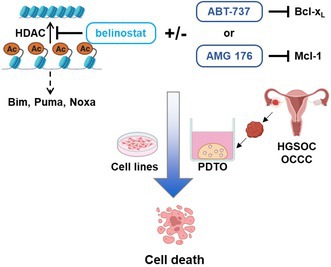

**Abbreviations**
H3K9: histone H3 lysine 9
HDAC: histone deacetylase
HGSOC: high‐grade serous ovarian carcinoma
OCCC: ovarian clear cell carcinoma
PDTO: patient‐derived tumor organoid

## 1 Introduction

Epithelial ovarian cancer remains the leading cause of death from gynecologic cancer in the United States [1]. Most patients are diagnosed late because early‐stage disease is usually asymptomatic. Despite a good initial response to standard treatment, which includes debulking surgery and platinum/taxane‐based chemotherapy [2], the majority of patients relapse and develop chemoresistance. As a result, the 5‐year relative survival rate is only 50% and decreases to approximately 30% when advanced‐stage patients are considered [1, 2]. The recent introduction of PARP inhibitors in the first‐line maintenance treatment setting has provided unprecedented benefits for ovarian cancer patients [3]. However, the efficacy of this targeted therapy is restricted to a subset of patients and may be compromised by the development of resistance. Thus, identifying other innovative strategies remains a major challenge to further improve the clinical management of ovarian cancer.

One of the hallmarks of cancer cells is their ability to evade apoptotic cell death. In particular, the overexpression of anti‐apoptotic proteins of the Bcl‐2 family is frequently implicated in carcinogenesis and chemoresistance [4], especially in ovarian cancers [5]. This deregulation disrupts the balance between anti‐ and pro‐apoptotic members of the Bcl‐2 family, leading to an excessive trapping of pro‐apoptotic proteins by their anti‐apoptotic partners. This prevents the activation of the multidomain proteins Bax and Bak by BH3‐only proteins, thereby inhibiting their oligomerization and subsequent permeabilization of the mitochondrial outer membrane [6]. More specifically, our previous work has shown that the survival of ovarian cancer cells relies on the cooperation between Bcl‐x_L_ and Mcl‐1 anti‐apoptotic proteins to sequester their pro‐apoptotic partners [7]. Beyond inhibiting Bcl‐x_L_ and Mcl‐1, increasing the expression levels of these pro‐apoptotic members, especially the BH3‐only proteins Bim, Puma, and Noxa, has proven to be an interesting strategy for triggering apoptosis in ovarian cancer cells [8, 9, 10].

The expression of genes encoding Bim, Puma, and Noxa can be restricted by epigenetic mechanisms. In particular, histone deacetylation, which increases chromatin compaction, may be responsible for repressing the transcription of these genes [11, 12, 13]. The homeostasis of histone acetylation is regulated by the activity of both histone acetyltransferase (HAT) and histone deacetylase (HDAC) enzymes, and may be disrupted by excessive HDAC expression or activity. Such HDAC aberrations have been implicated in carcinogenesis through various molecular mechanisms, particularly apoptosis inhibition [14]. Many tumors, including ovarian tumors, show overexpression of HDACs, which appears to be a poor prognostic factor [15, 16].

Several inhibitors, such as vorinostat, panobinostat, and belinostat, have been developed to target HDAC enzymes [14]. These molecules have proven effective in inhibiting tumor growth and inducing cancer cell apoptosis. Interestingly, they can increase the expression of pro‐apoptotic proteins of the Bcl‐2 family, such as Bim, Puma, Noxa, and Bax, while conversely repressing the expression of anti‐apoptotic members of this family, such as Bcl‐x_L_, Mcl‐1, and Bcl‐2 [17]. A few HDAC inhibitors have been approved by the Food and Drug Administration (FDA) for the treatment of certain hematological malignancies. These include belinostat (PXD101, Beleodaq^®^), a pan‐HDAC inhibitor used since 2014 to treat relapsed or refractory peripheral T‐cell lymphoma [18]. Preclinical studies have shown that it exerts anticancer effects, including in models of solid tumors such as pancreatic [19], prostate [20], lung [21], and thyroid cancer [22].

The objective of this study was to evaluate whether belinostat could be an interesting pharmacological tool to increase the expression of Bim, Puma, and/or Noxa, and to induce apoptosis in ovarian cancers, either alone or in combination with strategies targeting Bcl‐x_L_ or Mcl‐1.

## 2 Materials and methods

### 2.1 Cell culture and treatment

The human platinum‐resistant ovarian carcinoma cell lines SKOV3 (RRID:CVCL_0532) and IGROV1‐R10 were used. The SKOV3 cell line was obtained from the American Type Culture Collection. The IGROV1‐R10 cell line was established as described previously [23] from the IGROV1 cell line (RRID:CVCL_1304), that was kindly provided by Dr. Jean Bénard (Institut Gustave Roussy, Paris, France). These cell lines were authenticated in January 2024 by Microsynth (Balgach, Switzerland), who compared their short tandem repeat profiles with the Cellosaurus database. They were certified mycoplasma‐free using the MycoAlert test (Lonza, Basel, Switzerland). Cells were grown in RPMI 1640 + Glutamax and HEPES (Gibco, Paisley, UK), supplemented with 10% decomplemented Fetal Bovine Serum (Gibco) and 33 mm sodium bicarbonate (Gibco). They were maintained in a humidified atmosphere with 5% CO_2_ at 37 °C.

Belinostat and ABT‐737 were supplied by Selleckchem (Houston, TX, USA), and AMG 176 was provided by Chemietek (Indianapolis, IN, USA). Stock solutions were prepared in dimethyl sulfoxide (DMSO) and stored at −80 °C. A total of 2 × 10^5^ SKOV3 cells and 5.5 × 10^5^ IGROV1‐R10 cells were plated in 25‐cm^2^ flasks. Twenty‐four hours later, cells were treated with belinostat ± ABT‐737 or AMG 176. Control cells were exposed to the DMSO vehicle.

### 2.2 siRNA transfection

Bcl‐x_L_ siRNA, designated si‐Bcl‐x_L_ (siRNA antisense sequence: 5′‐auuggugagucggaucgcatt‐3′), and Mcl‐1 siRNA, designated si‐Mcl‐1 (siRNA antisense sequence: 5′‐gugccuuuguggcuaaacatt‐3′), were chemically synthesized by Eurogentec (Seraing, Belgium). SiGENOME non‐targeting control siRNA Pool#1 (SMARTpool), designated si‐Ctrl, was purchased from Dharmacon (Horizon Discovery, Cambridge, UK). All siRNAs were received as annealed oligonucleotides. A total of 1.5 × 10^5^ SKOV3 cells and 3.5 × 10^5^ IGROV1‐R10 cells were plated in 25‐cm^2^ flasks and transfected 24 h later. Briefly, the transfection reagent INTERFERin (Polyplus Transfection, Illkirch‐Graffenstaden, France) was added to siRNAs diluted in Opti‐MEM Reduced‐Serum Medium (Gibco). Complexes were allowed to form for 10 min at room temperature before they were applied to cells at a final concentration of 20 nm. Twenty‐four hours after transfection, the cell media was removed, and cells were treated with belinostat.

### 2.3 Analysis of cell proliferation and viability

Cell proliferation and viability were analyzed by studying the cell morphology by microscopy, the number of viable cells using the Trypan blue exclusion method, and the cellular DNA content by flow cytometry. For flow cytometry analysis, adherent and floating cells were pooled, washed with 1× Phosphate‐buffered saline (PBS) and fixed in 70% ethanol. Cells were then centrifuged at 2750 g for 5 min and incubated for 30 min at 37 °C in PBS. After centrifugation (2750 g for 5 min), cell pellets were dissociated and incubated with RNase A (Invitrogen, Waltham, MA, USA) and propidium iodide (Invitrogen). Samples were analyzed using a Gallios flow cytometer (Beckman Coulter) and both cell cycle distribution and sub‐G1 fraction were determined using Gallios software (Beckman Coulter, Brea, CA, USA).

### 2.4 RNA extraction and real‐time quantitative reverse transcription polymerase chain reaction (RT‐qPCR)

Total RNAs were extracted using TRIzol^®^ reagent (Invitrogen). RNA quantity and quality were assessed using a NanoDrop™ 2000 spectrophotometer (ThermoScientific, Waltham, MA, USA). RNA was reverse‐transcribed using Omniscript Reverse Transcription Kit (Qiagen, Venlo, Netherlands) and random primers (Invitrogen). The expression of the different transcripts was determined by real‐time quantitative PCR, with *GAPDH* (Glyceraldehyde‐3‐phosphate dehydrogenase) used as a housekeeping reference gene for normalization. To assess *BIM* and *PUMA* expression, the cDNAs were combined with the corresponding inventoried TaqMan™ Gene Expression Assays (Hs00708019_s1 for *BIM*, Hs00248075_m1 for *PUMA* and Hs99999905_m1 for *GAPDH*, Applied Biosystems, Carlsbad, CA, USA) and Taqman™ Universal Master Mix II, no UNG (Applied Biosystems). To assess *BCL‐X*_*L*_, *MCL‐1*, and *NOXA* expression, the cDNAs were combined with LightCycler^®^480 SYBR Green I Master PCR reaction mix (Roche, Basel, Switzerland), and forward and reverse primers (*BCL‐X*_*L*_ forward: 5′‐CCTTGGATCCAGGAGAACGG‐3′; *BCL‐X*_*L*_ reverse: 5′‐AAGAGTGAGCCCAGCAGAAC‐3′; *MCL‐1* forward: 5′‐TAACAAACTGGGGCAGGATT‐3′; *MCL‐1* reverse: 5′‐ATGGTTCGATGCAGCTTTCT‐3′; *NOXA* forward: 5′‐GACAAACTGAACTTCCGGCA‐3′; *NOXA* reverse: 5′‐ACGTGCACCTCCTGAGAAAA‐3′; *GAPDH* forward: 5′‐GAAAGCCTGCCGGTGACTAA‐3′; and *GAPDH* reverse: 5′‐AGGAAAAGCATCACCCGGAG‐3′, Eurogentec). All PCR amplification reactions were carried out in triplicate on a LightCycler^®^ 480 Real‐Time PCR instrument (Roche). The 2^−ΔΔCt^ method was used to calculate the relative changes in gene expression in treated cells as compared to control cells.

### 2.5 Extraction of proteins and western blot analysis

Proteins were extracted, and western blots were performed as described previously [9]. Acetyl‐Histone H3 (Lys9) (#9649), Caspase‐3 (#9662), PARP (#9542), Bim (#2819), Puma (#12450), Bcl‐x_L_ (#2764) and Mcl‐1 (#5453) antibodies were purchased from Cell Signaling Technology (Danvers, MA, USA), Noxa antibody (#OP180) from Calbiochem (Darmstadt, Germany), and α‐tubulin antibody (#T6199) from Sigma‐Aldrich (Darmstadt, Germany). Western blots shown are from one experiment representative of at least three independent experiments and cell lysates. Signals were quantified by pixel densitometry using the imagej software.

### 2.6 Patient‐derived tumor organoid (PDTO) experiments

#### 2.6.1 Tumor samples

Fresh tumor tissues were collected by the Biological Resources Center ‘OvaRessources’ (NF‐S 96900: 2016 quality management) from a patient with ovarian clear cell carcinoma (OCCC, anonymization number OV‐009_T) and from a patient with high‐grade serous ovarian carcinoma (HGSOC, anonymization number OV‐174_T), both of whom were treated at the Comprehensive Cancer Center François Baclesse (Unicancer Center, Caen, Normandy, France). The biological collection was declared to the French Ministry of Higher Education and Research (No. DC‐2020‐4221). Informed consent forms were signed by the patients and were obtained under the agreement of the ethical committee ‘North‐West III’ (CPP).

#### 2.6.2 PDTO establishment, culture, and treatment

PDTO were established, cultured, and treated as previously described [24] using Cultrex Reduced Growth Factor Basement Membrane Extract, Type 2 (Biotechne, Minneapolis, MN, USA) as extracellular matrix.

#### 2.6.3 Assessment of PDTO response to treatment

Real‐time monitoring of PDTO (morphology, size, and growth) was performed by the IncuCyte^®^ S3 (Essen BioScience, Ann Arbor, MI, USA). After a 48‐h treatment, cell viability was assessed by quantifying ATP levels using the CellTiter‐Glo^®^ 3D cell viability assay (Promega, Madison, WI, USA) and caspase‐3/7 activity was evaluated using the Caspase‐Glo^®^ 3/7 assay (Promega). Luminescence was measured using a Centro XS3 LB 960 (Berthold Technologies, Bad Wildbad, Germany) with Miko Win 2000 software. Viability and caspase‐3/7 activity curves were generated using GraphPad Prism software. Results were normalized to the DMSO control.

Immunohistochemical analysis was performed as follows: after a 48‐h treatment, PDTO were fixed in 3% paraformaldehyde for 4 h. They were then embedded in 2% agarose, dehydrated, embedded in paraffin, and sectioned before standard hematoxylin and eosin staining. Automated immunohistochemistry using a Ventana Discovery XT autostainer (Roche) was performed on 4 μm‐thick paraffin sections. After dewaxing, epitope unmasking, and inhibition of endogenous peroxidase activity, sections were incubated for 40 min at 37 °C with an anti‐cleaved caspase‐3 (Asp 175) antibody (#9661, Cell Signaling Technology). Sections were then rinsed with reaction buffer, and the secondary antibody (Omnimap Rabbit HRP, Roche) was added for 16 min at 37 °C. After washes, the staining was performed with 3,3′‐diaminobenzidine (DAB) and sections were counterstained with hematoxylin using Ventana reagents. Stained slides were digitized using a ScanScope CS scanner (Leica Biosystems, Nussloch, Germany).

### 2.7 Statistical analysis

The results were expressed as the mean ± SD (error bars) of at least three independent experiments for cell lines and at least two independent experiments for PDTO. Samples were compared using a one‐sample Student's *t*‐test. Differences were considered statistically different if *P* < 0.05 (*), *P* < 0.01 (**), or *P* < 0.001 (***).

## 3 Results

### 3.1 Belinostat exhibits a cytostatic or a cytotoxic effect, depending on the concentration used, in the SKOV3 and IGROV1‐R10 ovarian cancer cell lines

**Fig. 1:**
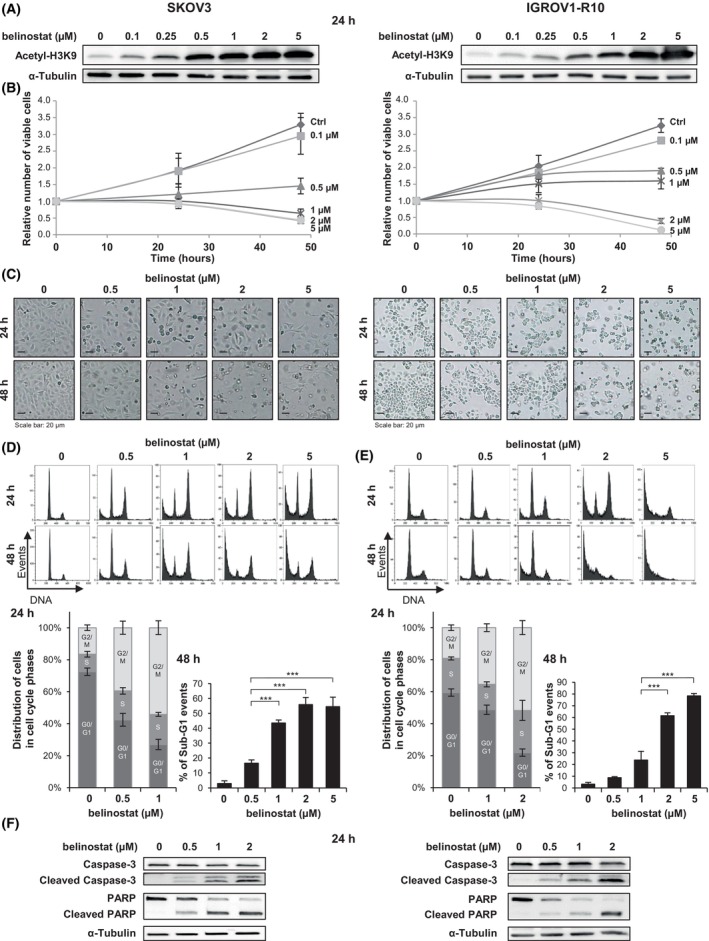
Belinostat exhibits a cytostatic or a cytotoxic effect, depending on the concentration used, in the SKOV3 and IGROV1‐R10 ovarian cancer cell lines. The effects of belinostat at various concentrations were analyzed in the ovarian cancer cell lines SKOV3 (left column) and IGROV1‐R10 (right column) at 24 h and 48 h. The level of histone H3 lysine 9 (H3K9) acetylation was assessed by western blot (A). Cell proliferation and apoptosis induction were studied by analyzing the relative number of viable cells using the trypan blue exclusion test (B), the cell morphology (scale bar: 20 μm) (C), and the DNA content histograms obtained by flow cytometry (D and E, top). The distribution of the cells in the cell cycle phases (D and E, bottom left) and the percentage of sub‐G1 events (D and E, bottom right) were deduced from these histograms. The results are expressed as the mean ± SD (error bars) of three independent experiments. Additionally, the cleavage of caspase‐3 and PARP was studied by western blot (F). ***P < 0.001 (Student's *t*‐test).

We analyzed the effect of belinostat as a single agent in the platinum‐resistant ovarian cancer cell lines SKOV3 and IGROV1‐R10. We first checked that treatment with this molecule increased the acetylation of histone H3 lysine 9 (H3K9), suggesting efficient inhibition of HDAC activity (Fig. 1A).

We showed that belinostat exhibited a cytostatic effect in SKOV3 and IGROV1‐R10 cells when used at concentrations of 0.5 μm and 1 μm, respectively. Treatment with these concentrations thus strongly limited the increase in the number of viable cells over time, as evidenced by both the growth curves (Fig. 1B) and the levels of cell confluency (Fig. 1C). This anti‐proliferative effect was associated with a cell cycle G2/M block at 24 h (Fig. 1D,E, top). Compared to the control, the relative number of cells in the G2/M phases increased 2.1‐fold in SKOV3 cells treated with 0.5 μm belinostat and 1.6‐fold in IGROV1‐R10 cells treated with 1 μm belinostat, and it further increased in response to higher concentrations (Fig. 1D,E, bottom left).

Moreover, belinostat induced apoptosis from 1 μm in the SKOV3 cell line and from 2 μm in the IGROV1‐R10 cell line. Indeed, treatment with these concentrations led to a reduction in the number of viable cells over time (Fig. 1B), which was associated with cell detachment (Fig. 1C) and the emergence of a high sub‐G1 peak (Fig. 1D,E, top). Specifically, the percentage of sub‐G1 events reached 44% in SKOV3 cells and 61% in IGROV1‐R10 cells after a 48‐h treatment with 1 μm and 2 μm belinostat, respectively (Fig. 1D,E, bottom right). Finally, the induction of apoptotic cell death in response to these concentrations was confirmed by the detection of both caspase‐3 and PARP cleavage (Fig. 1F).

Altogether, these results show that belinostat exerts anti‐proliferative and pro‐apoptotic effects in both cell lines, with SKOV3 cells appearing to be more sensitive than IGROV1‐R10 cells.

### 3.2 Belinostat induces Bim and Puma expression and partially inhibits that of Bcl‐x_L_ , even at sub‐apoptotic concentration

**Fig. 2:**
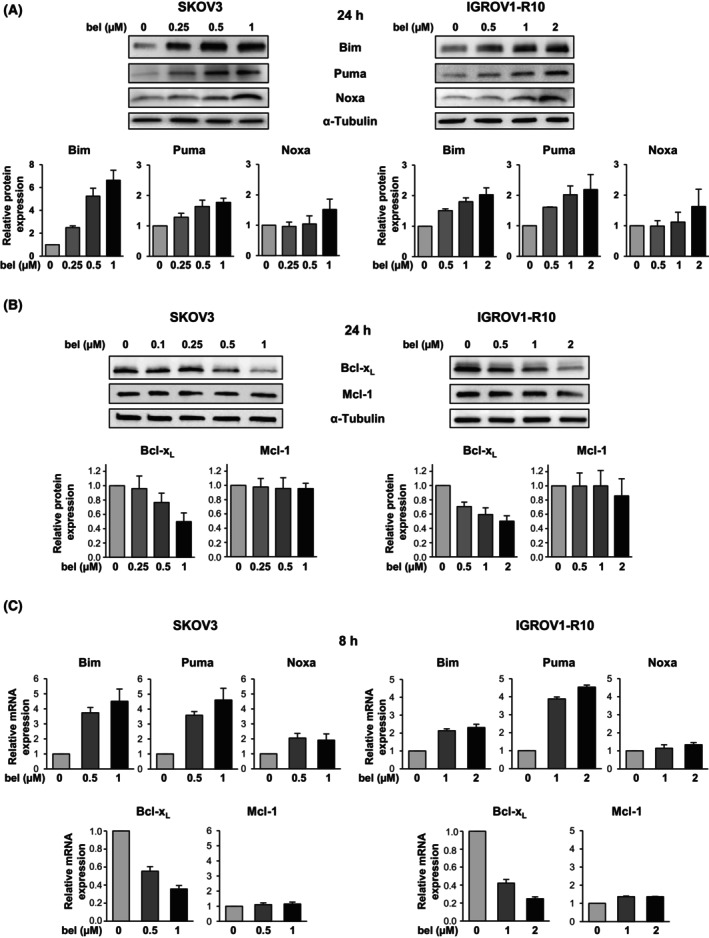
Belinostat induces Bim and Puma expression and partially inhibits that of Bcl‐x_L_, even at sub‐apoptotic concentration. The effect of belinostat (bel) at various concentrations on the expression of pro‐apoptotic proteins Bim, Puma, and Noxa (A) and anti‐apoptotic proteins Bcl‐x_L_ and Mcl‐1 (B) in the SKOV3 and IGROV1‐R10 cell lines was investigated by western blot at 24 h. The expression levels of these proteins were quantified using ImageJ software and normalized to that of α‐tubulin. Histograms represent the relative protein expression in the treated cells normalized to that of control cells. The results are expressed as the mean ± SD (error bars) of three (A) or four (B) independent experiments. The effect of belinostat at various concentrations on the mRNA expression of Bim, Puma, Noxa, Bcl‐x_L_, and Mcl‐1 in the SKOV3 and IGROV1‐R10 cell lines was assessed by RT‐qPCR at 8 h (C). *GAPDH* was used as a housekeeping reference gene for normalization. Histograms represent the relative mRNA expression in the treated cells normalized to that of control cells. The results are expressed as the mean ± SD (error bars) of three (for Noxa and Mcl‐1) or four (for Bim, Puma and Bcl‐x_L_) independent experiments.

We then explored the effect of belinostat on the expression of the Bcl‐2 family proteins that have previously been described as crucial for regulating the apoptosis of ovarian cancer cells [7, 8, 9, 10]. A 24‐h treatment with belinostat induced a concentration‐dependent increase in Bim and Puma protein expression in both cell lines (Fig. 2A). Interestingly, this upregulation was observed even in response to the cytostatic concentration (0.5 μm in the SKOV3 cell line and 1 μm in the IGROV1‐R10 cell line). A slight increase in Noxa protein expression was also detected, but only in response to the apoptotic concentration (1 μm in SKOV3 cells and 2 μm in IGROV1‐R10 cells). Furthermore, belinostat elicited a concentration‐dependent decrease in Bcl‐x_L_ protein expression, with the strongest inhibition (50%) observed at the apoptotic concentration (Fig. 2B). In contrast, belinostat treatment had little impact on Mcl‐1 protein expression. It is noteworthy that the variations in the protein expression of Bim, Puma, Noxa, and Bcl‐x_L_ in response to belinostat were accompanied by corresponding variations in the expression of their respective mRNAs (Fig. 2C).

As Bim, Puma, and Noxa proteins are upregulated in response to the apoptotic concentration of belinostat, we aimed to determine whether they could play a role in its cytotoxic effect. Using siRNAs, we inhibited the expression of each of these proteins in SKOV3 and IGROV1‐R10 cells before exposing them to the apoptotic concentration of belinostat (Fig. S1A). In SKOV3 cells, assessment of both the relative number of viable cells and the percentage of sub‐G1 events suggested that inhibition of Noxa protected cells from belinostat‐induced apoptosis (Fig. S1B, left panel). This was confirmed by the increased cell confluency and the absence of caspase‐3 cleavage observed in the treated cells in which Noxa had been silenced (Fig. S1C,D, left panel). In IGROV1‐R10 cells, our results collectively suggested that Bim might play a role in the apoptotic effect of belinostat, as inhibition of its expression partially protected cells from death (Fig. S1B–D, right panel).

Since belinostat used at cytostatic concentration increased the expression of Bim and Puma while partially repressing that of Bcl‐x_L_, we hypothesized that combining it with strategies inhibiting residual Bcl‐x_L_ or Mcl‐1 proteins might be effective in inducing apoptosis in ovarian cancer cells.

### 3.3 Bcl‐x_L_ inhibition sensitizes the SKOV3 and IGROV1‐R10 ovarian cancer cell lines to belinostat

**Fig. 3:**
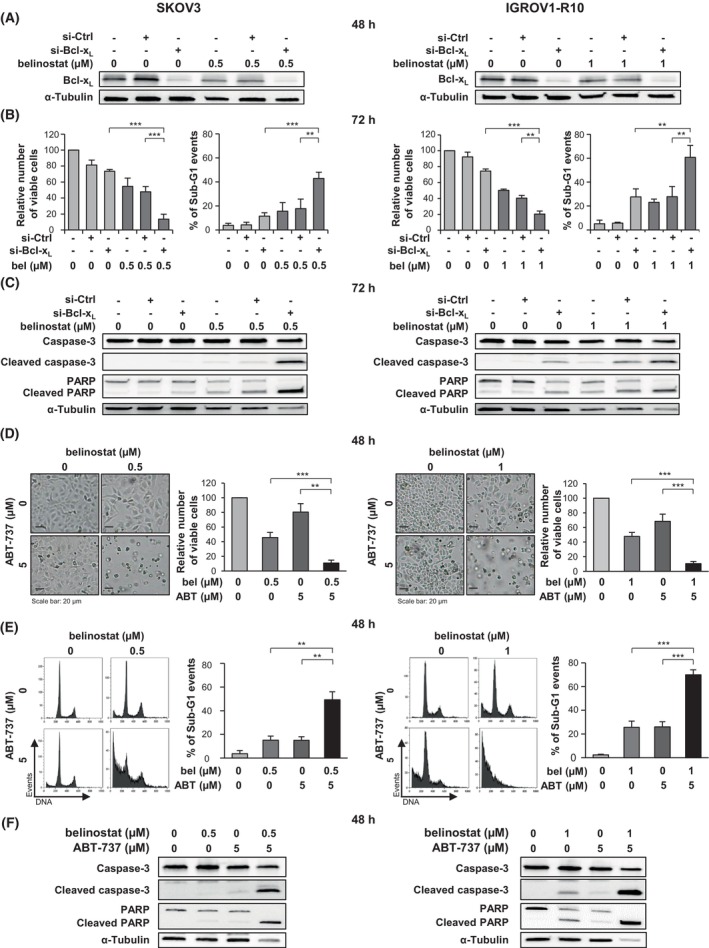
Bcl‐x_L_ inhibition sensitizes the SKOV3 and IGROV1‐R10 ovarian cancer cell lines to belinostat. SKOV3 (left column) and IGROV1‐R10 (right column) cells were transfected with Control (si‐Ctrl) or Bcl‐x_L_ (si‐Bcl‐x_L_) siRNAs and treated 24 h later with belinostat (bel) at cytostatic concentration (A–C). The efficacy of Bcl‐x_L_ silencing was monitored by western blot 48 h after transfection (A). The impact of combining belinostat treatment with Bcl‐x_L_ inhibition was investigated at 72 h by analyzing the relative number of viable cells using the trypan blue exclusion test and the percentage of sub‐G1 events obtained by flow cytometry (B), as well as by studying the cleavage of caspase‐3 and PARP by western blot (C). SKOV3 (left column) and IGROV1‐R10 (right column) cells were treated with both belinostat at cytostatic concentration and the Bcl‐x_L_ inhibitor ABT‐737 at 5 μm for 48 h (D–F). The efficacy of the co‐treatment was explored by analyzing the cell morphology (scale bar: 20 μm) and the relative number of viable cells using the trypan blue exclusion test (D), the DNA content histograms and the percentage of sub‐G1 events, both obtained by flow cytometry (E), and the cleavage of caspase‐3 and PARP detected by western blot (F). The results shown in the graphs are expressed as the mean ± SD (error bars) of four (B) or three (D and E) independent experiments. **P < 0.01; ***P < 0.001 (Student's *t*‐test).

We first investigated whether silencing Bcl‐x_L_ expression using siRNA could potentiate the effect of belinostat when used at cytostatic concentration in SKOV3 and IGROV1‐R10 cells. We checked that the expression of this anti‐apoptotic protein was repressed 48 h after transfection with si‐Bcl‐x_L_, whether or not the cells were treated with belinostat (Fig. 3A). Interestingly, inhibiting Bcl‐x_L_ expression sensitized SKOV3 and IGROV1‐R10 cells to belinostat, resulting in the induction of apoptotic cell death. This was evidenced by the decrease in the number of viable cells and the concomitant increase in the percentage of sub‐G1 events observed in the si‐Bcl‐x_L_/belinostat condition, compared to both the si‐Bcl‐x_L_/DMSO and si‐Ctrl/belinostat conditions (Fig. 3B). This was also supported by the detection of a significant cleavage of caspase‐3 and PARP in the si‐Bcl‐x_L_/belinostat condition (Fig. 3C).

The activity of Bcl‐x_L_ can be inhibited using BH3‐mimetic molecules, such as ABT‐737, which binds with high affinity to the hydrophobic groove of Bcl‐x_L_, Bcl‐2, and Bcl‐w and antagonizes their anti‐apoptotic function [25]. We then evaluated the impact of a treatment combining belinostat at cytostatic concentration with ABT‐737 at 5 μm for 48 h. In contrast to treatment with either of these molecules alone, the co‐treatment triggered major cell detachment in both cell lines, accompanied by a drastic reduction in the number of viable cells (Fig. 3D). This was the consequence of a strong induction of apoptosis, as supported by the high sub‐G1 peak (Fig. 3E) and marked cleavage of caspase‐3 and PARP (Fig. 3F). The percentage of sub‐G1 events in response to the combination treatment reached 49% in SKOV3 cells (compared to approximately 15% in response to belinostat or ABT‐737 alone, Fig. 3E, left panel) and 70% in IGROV1‐R10 cells (compared to approximately 25% in response to belinostat or ABT‐737 alone, Fig. 3E, right panel). A similar apoptotic effect was obtained by combining belinostat with a selective inhibitor of Bcl‐x_L_, A‐1331852 (Fig. S2A–C) [26].

To conclude, inhibiting Bcl‐x_L_ expression or activity constitutes a relevant strategy to enhance the anticancer effect of belinostat in both SKOV3 and IGROV1‐R10 cells.

### 3.4 Mcl‐1 inhibition sensitizes the IGROV1‐R10 ovarian cancer cell line to belinostat

**Fig. 4:**
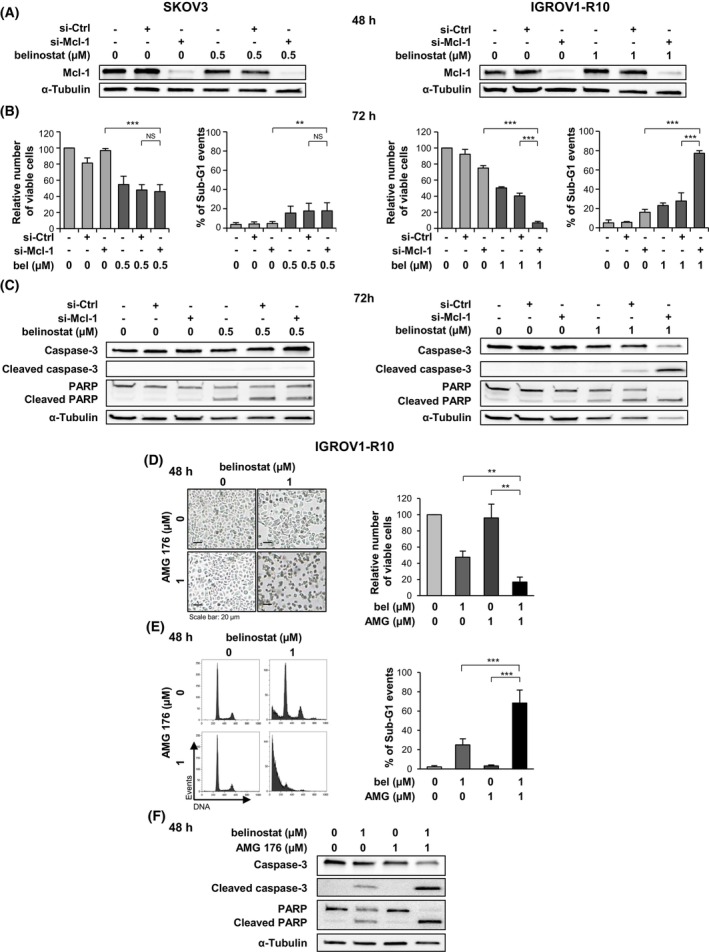
Mcl‐1 inhibition sensitizes the IGROV1‐R10 ovarian cancer cell line to belinostat. SKOV3 (left column) and IGROV1‐R10 (right column) cells were transfected with Control (si‐Ctrl) or Mcl‐1 (si‐Mcl‐1) siRNAs and treated 24 h later with belinostat (bel) at cytostatic concentration (A–C). The efficacy of Mcl‐1 silencing was monitored by western blot 48 h after transfection (A). The impact of combining belinostat treatment with Mcl‐1 inhibition was investigated at 72 h by analyzing the relative number of viable cells using the trypan blue exclusion test and the percentage of sub‐G1 events obtained by flow cytometry (B), as well as by studying the cleavage of caspase‐3 and PARP by western blot (C). IGROV1‐R10 cells were treated with both belinostat at cytostatic concentration and the Mcl‐1 inhibitor AMG 176 at 1 μm for 48 h (D–F). The efficacy of the co‐treatment was explored by analyzing the cell morphology (scale bar: 20 μm) and the relative number of viable cells using the trypan blue exclusion test (D), the DNA content histograms and the percentage of sub‐G1 events, both obtained by flow cytometry (E), and the cleavage of caspase‐3 and PARP detected by western blot (F). The results shown in the graphs are expressed as the mean ±SD (error bars) of four (B) or three (D and E) independent experiments. NS: not significant; **P < 0.01; ***P < 0.001 (Student's *t*‐test).

Our next objective was to explore the impact of Mcl‐1 inhibition on the response of SKOV3 and IGROV1‐R10 cells to belinostat used at cytostatic concentration. We first repressed Mcl‐1 protein expression using siRNA (Fig. 4A). In the SKOV3 cell line, Mcl‐1 silencing did not modify the response of cells to 0.5 μm belinostat (Fig. 4B,C, left panels). In contrast, inhibiting Mcl‐1 expression effectively sensitized IGROV1‐R10 cells to belinostat used at cytostatic concentration. Cells that were both transfected with si‐Mcl‐1 and treated with belinostat exhibited a significant decrease in the number of viable cells and a substantial accumulation of sub‐G1 events (Fig. 4B, right panel), as well as more pronounced cleavage of caspase‐3 and PARP (Fig. 4C, right panel), compared to the si‐Mcl‐1/DMSO and si‐Ctrl/belinostat conditions.

We then sought to confirm these results using AMG 176, a BH3‐mimetic compound that inhibits Mcl‐1 activity with high affinity and selectivity [27], in IGROV1‐R10 cells. The treatment combining belinostat (1 μm) and AMG 176 (1 μm) for 48 h proved to be highly effective in inducing apoptosis in these cells. This co‐treatment led to a loss of the adherent cell layer, with the relative number of viable cells decreasing to 17% (Fig. 4D), while dramatically increasing the presence of apoptotic bodies, with the percentage of sub‐G1 events amounting to 68% (Fig. 4E). The significant cleavage of caspase‐3 and PARP observed in response to the belinostat/AMG 176 combination further confirmed its efficacy (Fig. 4F). On the contrary, at the concentrations used, neither AMG 176 nor belinostat alone exhibited a major apoptotic effect (Fig. 4D–F).

Altogether, these results highlight that Mcl‐1 inhibition sensitizes IGROV1‐R10 cells, but not SKOV3 cells, to belinostat.

### 3.5 Belinostat shows anticancer effects in PDTO models, which are enhanced by inhibitors of Bcl‐x_L_ or Mcl‐1

Our final objective was to validate our results using models that more accurately reproduce the clinical situation than cell lines. Therefore, we assessed the impact of belinostat as a single agent or in combination with inhibitors of Bcl‐x_L_ or Mcl‐1 in patient‐derived tumor organoid models.

**Fig. 5:**
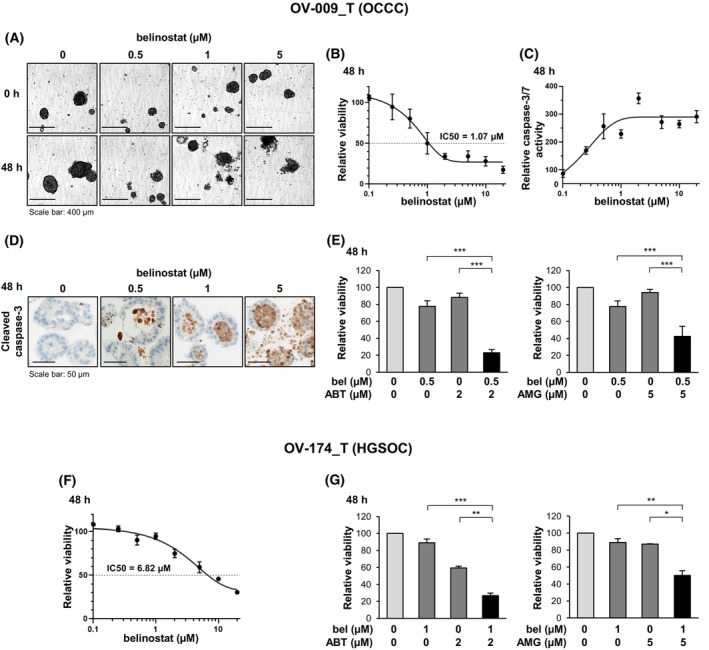
Belinostat shows anticancer effects in PDTO models, which are enhanced by inhibitors of Bcl‐x_L_ or Mcl‐1. The OV‐009_T PDTO model, established from an OCCC, was treated for 48 h with belinostat at various concentrations (A–D). The impact of belinostat on the PDTO was investigated by analyzing their morphology (scale bar: 400 μm) (A), their viability, assessed by the CellTiter‐Glo®3D cell viability assay (B), the activity of caspase‐3/7, assessed by the Caspase‐Glo^®^ 3/7 assay (C) and the cleavage of caspase‐3 detected by immunohistochemistry (scale bar: 50 μm) (D). The OV‐009_T PDTO were co‐treated for 48 h with belinostat (bel) at 0.5 μm (low‐toxic concentration) and either the Bcl‐x_L_ inhibitor ABT‐737 at 2 μm (E, left panel) or the Mcl‐1 inhibitor AMG 176 at 5 μm (E, right panel). The effect of these combinations was analyzed by evaluating the viability of the PDTO using the CellTiter‐Glo^®^ 3D cell viability assay. The OV‐174_T PDTO model, established from an HGSOC, was either treated for 48 h with belinostat at various concentrations (F) or co‐treated with belinostat at 1 μm (low‐toxic concentration) and either ABT‐737 at 2 μm (G, left panel) or AMG 176 at 5 μm (G, right panel). The effect of the treatments was analyzed by evaluating the viability of the PDTO using the CellTiter‐Glo^®^ 3D cell viability assay. The results of the graphs shown in (E) and (G) are expressed as the mean ± SD (error bars) of four and two independent experiments, respectively. *P < 0.05; **P < 0.01; ***P < 0.001 (Student's *t*‐test).

We initially studied the effect of a 48‐h treatment with belinostat at different concentrations in a PDTO model established from an ovarian clear cell carcinoma, designated OV‐009_T model. Belinostat caused the alteration of the PDTO structures starting at 1 μm (Fig. 5A) and led to a concentration‐dependent decrease in their viability (IC50 = 1.07 μm) (Fig. 5B). These effects were correlated with the induction of apoptosis, as supported by the increase in caspase‐3/7 activity in response to treatment (Fig. 5C) and the emergence of caspase‐3 cleavage detected by immunohistochemistry (Fig. 5D). We then combined belinostat at a concentration that weakly impacted viability (0.5 μm) with inhibitors of Bcl‐x_L_ (ABT‐737) or Mcl‐1 (AMG 176). Interestingly, both ABT‐737 and AMG 176 sensitized OV‐009_T PDTO to belinostat, as demonstrated by the significant decrease in their viability in response to the combined treatments (23% and 42% viability in response to belinostat/ABT‐737 and belinostat/AMG 176 combinations, respectively), compared to each molecule alone (78%, 88% and 94% viability in response to belinostat, ABT‐737 and AMG 176, respectively; Fig. 5E). Similar results were obtained when ABT‐737 was replaced with the selective inhibitor of Bcl‐x_L_ A‐1331852, confirming that the sensitizing effect of ABT‐737 can be attributed to Bcl‐x_L_ inhibition (Fig. S2D).

We finally investigated the effect of belinostat in a second PDTO model, OV‐174_T, derived from a high‐grade serous ovarian carcinoma. It was revealed to be less sensitive to belinostat than the OV‐009_T model (IC50 = 6.82 μm) (Fig. 5F). However, both ABT‐737 and AMG 176 also efficiently sensitized this PDTO model to belinostat (Fig. 5G).

Collectively, the results obtained in two PDTO models derived from OCCC or HGSOC confirm our previous findings, showing that belinostat displays anticancer properties, which are enhanced in the presence of Bcl‐x_L_ or Mcl‐1 inhibitors.

## 4 Discussion

Despite the recent progress brought by the development of PARP inhibitors, the implementation of targeted therapies for the treatment of ovarian carcinoma has lagged behind compared to other malignancies. This partly explains why the prognosis of this pathology remains poor and emphasizes the need to identify new therapeutic strategies.

Our previous work has shown that in ovarian cancer cells, the threshold for triggering apoptosis can be reached by reducing the [Bcl‐x_L_ and Mcl‐1]/[Bim, Puma, and Noxa] ratio. Indeed, we initially demonstrated that simultaneous inhibition of the expression of Bcl‐x_L_ and Mcl‐1 anti‐apoptotic proteins using siRNA induced massive cell death in ovarian cancer cell lines [7]. Furthermore, we found that upregulating the expression of their pro‐apoptotic BH3‐only partners Bim, Puma, and Noxa, using a MEK inhibitor combined with an mTOR inhibitor or an α_1_‐adrenergic receptor antagonist, was also accompanied by apoptosis induction [8, 10]. Since the expression of these pro‐apoptotic proteins can be hindered by histone deacetylation [11, 12, 13], the objective of this study was to assess the efficacy of belinostat, a clinically used HDAC inhibitor, in increasing their expression levels and inducing apoptotic cell death in ovarian cancers.

We first investigated the effect of belinostat in the platinum‐resistant ovarian cancer cell lines SKOV3 and IGROV1‐R10. Our results showed that belinostat exerted (i) a cytostatic effect at low concentration, associated with a G2/M block, and (ii) an apoptotic effect at higher concentrations. The SKOV3 cell line appeared to be more sensitive to this HDAC inhibitor than the IGROV1‐R10 cell line. These results converge with those of two previous studies that were mainly conducted in the A2780 ovarian cancer cell line. Belinostat inhibited the growth of A2780 cells when cultured *in vitro* and xenografted *in vivo* [28, 29], and induced apoptosis *in vitro*, albeit at very high concentrations (10‐fold higher than the IC50) [28].

To validate the therapeutic interest of belinostat in a preclinical model of ovarian cancer that exhibits greater clinical relevance than traditional 2D cell lines, we chose patient‐derived tumor organoids. These three‐dimensional models offer the advantage of recapitulating histological and genomic features of the tumors from which they are derived [30]. Moreover, their response to treatment has been shown to correlate with patients' clinical response [24, 31]. Consequently, they are emerging as the new benchmark for preclinical models, with great potential in precision medicine in oncology [32]. We studied the impact of belinostat on PDTO derived from two ovarian tumors with different histologic subtypes: one from OCCC, a rare form of ovarian cancer that exhibits a drug‐resistant phenotype, and the other from HGSOC, the most common type of ovarian cancer. Interestingly, belinostat exhibited anticancer effects in the OCCC‐derived model (OV‐009_T model), consistent with our previous findings [24], as well as in the HGSOC‐derived model (OV‐174_T model). The most sensitive of the two models was OV‐009_T. In these PDTO, the decrease in viability correlated with the induction of apoptosis, as evidenced by the increase in caspase‐3/7 activity and the detection of caspase‐3 cleavage by immunohistochemistry. Cleaved caspase‐3 was observed to localize in the center of the PDTO rather than in the outer matrix‐attached cell layer, as described elsewhere [33]. This could be explained by pro‐survival signals emanating from the extracellular matrix which may counteract the apoptotic signals. The anticancer effect of belinostat has previously been assessed on another 3D model of ovarian cancer – organoids generated from HGSOC patient‐derived cell lines – in a study that screened 22 clinically relevant drugs or drug combinations [34]. Using an automated microscopy‐based assay to assess drug‐induced cell death and proliferation inhibition, the authors highlighted that belinostat was one of the most effective therapeutic agents. However, the efficacy of belinostat appeared to be fairly modest in a phase II clinical trial, although this molecule was well tolerated, with fatigue and nausea representing the most common side effects [35]. In this clinical trial conducted in patients with platinum‐resistant recurrent ovarian cancer, stable disease was observed in 9 out of 15 cases. Identifying predictive markers of the response to belinostat and/or combining it with other molecules could improve its clinical efficacy. The association of belinostat with carboplatin and paclitaxel has demonstrated clinical benefit in heavily pretreated patients with ovarian cancer [36].

As expected, belinostat increased Bim, Puma, and Noxa protein expression in our ovarian cancer cell lines. Belinostat‐induced upregulation of Bim protein expression has also been reported in leukemia [37] and lymphoma cell lines [38], as well as in models of lung carcinoma [39], while the induction of Noxa protein in response to this HDAC inhibitor has been described in a lymphoma cell line [40]. However, to our knowledge, the upregulation of Puma protein expression in response to belinostat had never been documented before. Belinostat also partially decreased Bcl‐x_L_ protein expression in SKOV3 and IGROV1‐R10 cells, as previously observed in models of leukemia [37], pancreatic cancer [19], and prostate cancer [20]. The changes in the expression levels of Bim, Puma, Noxa, and Bcl‐x_L_ proteins were associated with corresponding changes in the expression levels of their respective mRNAs, suggesting the involvement of a transcriptional mechanism. The concentration of belinostat that resulted in the lowest [Bcl‐x_L_ and Mcl‐1]/[Bim, Puma, and Noxa] ratio (1 μm in SKOV3 cells and 2 μm in IGROV1‐R10 cells) was the one that triggered apoptotic cell death. Noteworthy, belinostat‐induced Noxa and Bim were found to play a role in this apoptosis in SKOV3 and IGROV1‐R10 cells, respectively. However, both the upregulation of Bim and Puma expression and the partial inhibition of Bcl‐x_L_ expression were already observable in response to the cytostatic concentration of belinostat, albeit to a lesser extent. We hypothesized that under this condition, Bim and Puma remained buffered by Mcl‐1 and/or residual Bcl‐x_L_, thereby preventing apoptosis. Consequently, we investigated the impact of combining belinostat at the cytostatic concentration with strategies inhibiting Bcl‐x_L_ or Mcl‐1.

Inhibiting Bcl‐x_L_ using siRNA or the BH3‐mimetics ABT‐737 or A‐1331852 was highly effective in sensitizing ovarian cancer cell lines to belinostat. Previous studies have reported the efficacy of the association of other HDAC inhibitors, such as vorinostat, with ABT‐737 or its orally available derivative ABT‐263, in preclinical models of hematological cancers [41, 42, 43, 44] and solid tumors [45, 46, 47]. However, such associations had never been assessed in preclinical models of ovarian cancer before. Moreover, our study provides the first evidence of the anticancer potential of combining the HDAC inhibitor belinostat with a Bcl‐x_L_ inhibitor. Interestingly, the efficacy of belinostat/ABT‐737 co‐treatment was confirmed in our two PDTO models, regardless of (i) the histologic subtype of the tumors from which they were established (HGSOC or OCCC) and (ii) their sensitivity to belinostat. Replacing ABT‐737 with A‐1331852 did not alter the combination efficacy, indicating that the sensitizing effect of ABT‐737 is indeed due to Bcl‐x_L_ inhibition. This original evaluation of the combination of an HDAC inhibitor with a Bcl‐x_L_ inhibitor in a 3D preclinical model that recapitulates the characteristics of the patient's solid tumor further argues in favor of the clinical potential of such a strategy.

Inhibition of Mcl‐1 has been shown to potentiate the anticancer effect of an HDAC inhibitor (panobinostat) in models of hematological cancer [48, 49]. However, the efficacy of such a strategy remained to be explored in models of solid tumors. This seemed particularly relevant as resistance to the HDAC inhibitor vorinostat had been reported to depend on the phosphorylation level of Mcl‐1 in colon cancer models [50]. We first explored the apoptotic potential of combining Mcl‐1 inhibition with belinostat at cytostatic concentration in our ovarian cancer cell lines. Silencing Mcl‐1 expression using siRNA was sufficient to lower the apoptotic threshold enough to sensitize IGROV1‐R10 cells to belinostat. In concordance, the combination of belinostat with the BH3‐mimetic AMG 176 induced massive cell death in this cell line. In contrast, Mcl‐1 inhibition did not sensitize SKOV3 cells to belinostat. Our previous data showed that in the basal state, SKOV3 cells displayed an approximately 4‐fold higher Bcl‐x_L_/Mcl‐1 protein expression ratio than IGROV1‐R10 cells, due to a lower level of Mcl‐1 expression [10]. The difference in this protein ratio between the cell lines was maintained in response to belinostat, as this molecule reduced Bcl‐x_L_ expression quite similarly in both cell lines and did not modulate Mcl‐1 expression. Therefore, it can be hypothesized that SKOV3 cells are more dependent on Bcl‐x_L_ than on Mcl‐1 for their survival compared to IGROV1‐R10 cells, even in response to belinostat. However, in our two PDTO models, the belinostat/AMG 176 combination displayed effective anticancer activity, supporting the concept that Mcl‐1 inhibition would represent a promising strategy to sensitize ovarian tumors to belinostat.

## 5 Conclusions

To conclude, our study underlines the anticancer potential of belinostat, used alone or combined with Bcl‐x_L_ or Mcl‐1 inhibitors, in preclinical models of ovarian cancer. In particular, demonstrating the efficacy of these strategies in PDTO established from ovarian carcinomas with different histologic subtypes emphasizes their clinical relevance. While belinostat is an FDA‐approved therapeutic molecule, the clinical use of BH3‐mimetic molecules targeting Bcl‐x_L_ or Mcl‐1 is currently limited due to the on‐target toxicity they cause to platelets and cardiomyocytes, respectively. One can expect that using these molecules in combination with belinostat could allow for a reduction of their dose to a level where their toxicity becomes manageable. Alternatively, approaches for inhibiting Bcl‐x_L_ or Mcl‐1 while avoiding thrombocytopenia or cardiac toxicity include the use of proteolysis‐targeting chimeras (PROTAC) that cause degradation of their target in a tissue‐specific manner [51, 52] or antibody‐drug conjugates (ADC) that selectively deliver the inhibitor to the tumor cells [53]. Our study thus paves the way for the development of belinostat‐based therapeutic strategies for ovarian cancer, with predictive biomarkers of response still to be identified.

## Conflict of interest

The authors have no conflicts of interest to declare.

## Author contributions

CT and SG carried out the cell line and PDTO experiments and analyzed the data; LBW and RF established the PDTO and supervised the PDTO experiments; EA provided technical support for the cell line experiments; FG carried out the immunohistochemistry experiments; EB performed real‐time monitoring of the PDTO; MB and ED provided the tumor samples from which the PDTO were established; LP supervised the study; MV designed and supervised the study, analyzed the data, and wrote the manuscript. All authors have read and approved the manuscript.

## Supporting information


**Fig. S1.** Noxa and Bim play a role in belinostat‐induced apoptosis in SKOV3 and IGROV1‐R10 cells, respectively.

**Fig. S2.** Bcl‐x_L_ selective inhibition with A‐1331852 sensitizes preclinical models of ovarian cancer to belinostat.

## Data Availability

The data that support the findings of this study are available within the article and its supplementary data files.
