# Supplementary material for: The anticancer effect of the HDAC inhibitor belinostat is enhanced by inhibitors of Bcl‐xL or Mcl‐1 in ovarian cancer
Source: Mol Oncol. 2025 Jun 8;19(11):3325–41. doi: 10.1002/1878-0261.70050 (PMC12591309; doi:10.1002/1878-0261.70050)
Supplement: Supplementary file 1 — Fig. S1. Noxa and Bim play a role in belinostat‐induced apoptosis in SKOV3 and IGROV1‐R10 cells, respectively. [file MOL2-19-3325-s001.pdf]

Supplementary figure S1, Thomine et al.

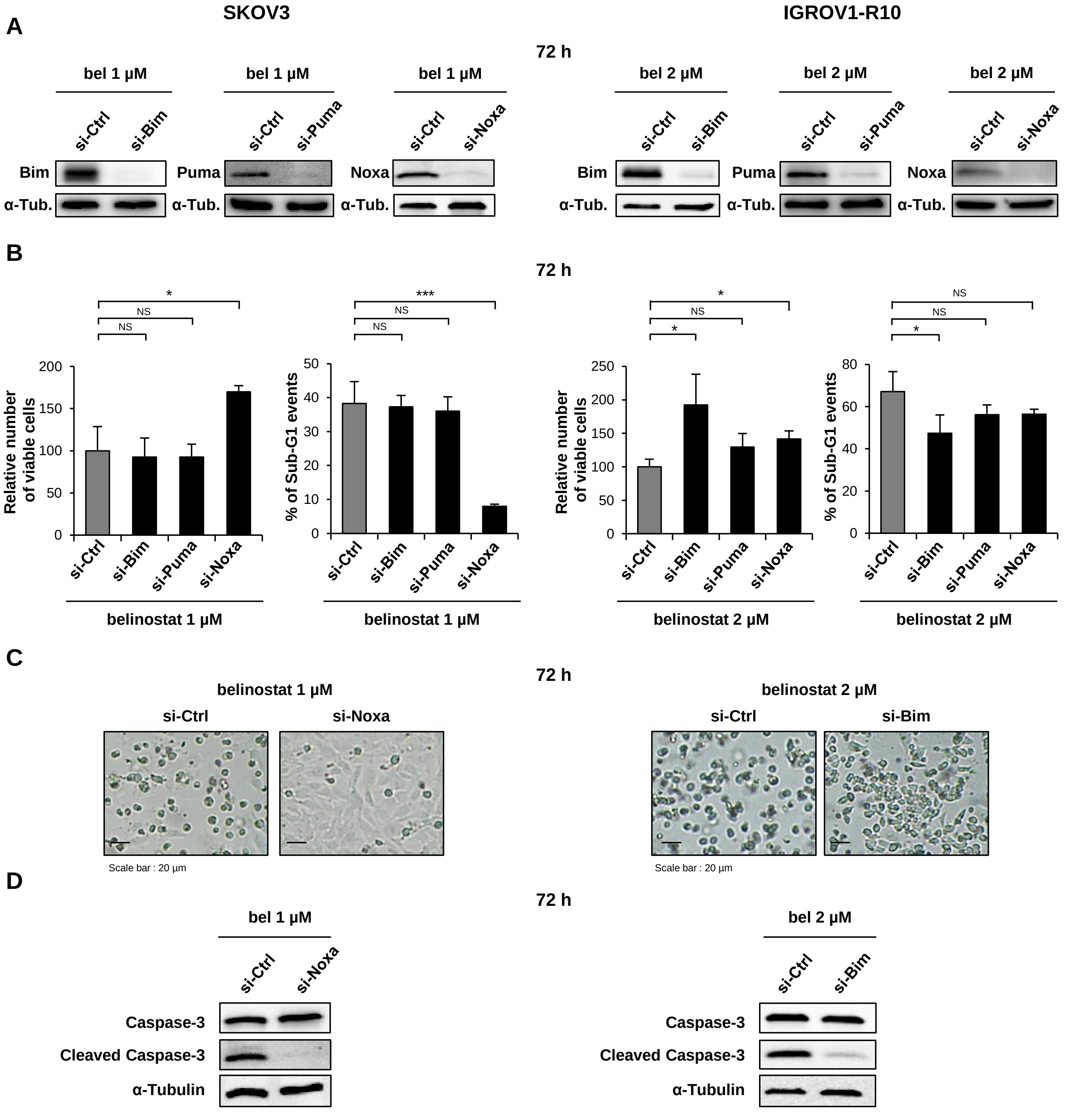

Noxa and Bim play a role in belinostat-induced apoptosis in SKOV3 and IGROV1-R10 cells, respectively. SKOV3 (left column) and IGROV1-R10 (right column) cells were transfected with Control (si-Ctrl), Bim (si-Bim), Puma (si-Puma) or Noxa (si-Noxa) siRNAs and treated 24 hours later with belinostat (bel) at the apoptotic concentration. The efficacy of Bim, Puma and Noxa silencing was monitored by western blot 72 hours after transfection (A). The impact of inhibiting the expression of these proteins on the cell response to belinostat was investigated at 72 h by analyzing the relative number of viable cells using the trypan blue exclusion test and the percentage of sub-G1 events assessed by flow cytometry (B), the cell morphology (scale bar: 20 µm) (C), and the cleavage of caspase-3 detected by western blot (D). The results shown in the graphs are expressed as the mean +/- SD (error bars) of three independent experiments. *p<0.05; ***p<0.001 (Student's t-test)

Materials and Methods for supplementary figure S1, Thomine *et al.*

siRNA transfection

Bim siRNA, designated si-Bim (siRNA antisense sequence: 5′-uaacagucguaagauaacctt-3′) and Puma siRNA, designated si-Puma (siRNA antisense sequence: 5′-uauacaguaucuuacaggctt-3′) were chemically synthesized by Eurogentec. Noxa siRNA, designated si-Noxa (SMARTpool), and ON-TARGETplus Non-Targeting control siRNA #1 (SMARTpool), designated si-Ctrl, were purchased from Dharmacon (Horizon Discovery). The transfection protocol is described in the 'Materials and Methods' section.
