# Supplementary material for: The anticancer effect of the HDAC inhibitor belinostat is enhanced by inhibitors of Bcl‐xL or Mcl‐1 in ovarian cancer
Source: Mol Oncol. 2025 Jun 8;19(11):3325–41. doi: 10.1002/1878-0261.70050 (PMC12591309; doi:10.1002/1878-0261.70050)
Supplement: Supplementary file 2 — Fig. S2. Bcl‐xL selective inhibition with A‐1331852 sensitizes preclinical models of ovarian cancer to belinostat. [file MOL2-19-3325-s002.pdf]

Supplementary figure S2, Thomine et al.

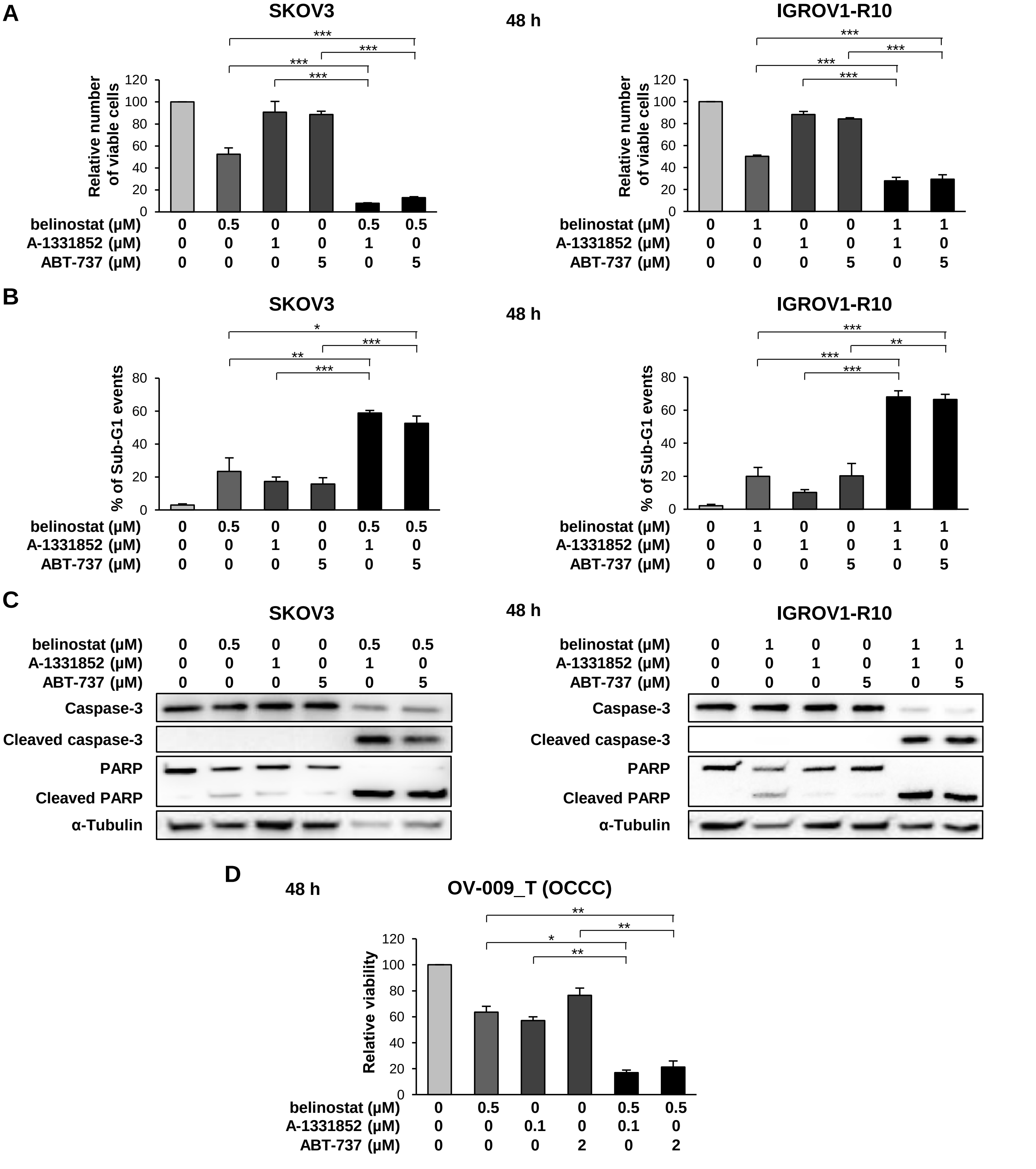

Bcl-x_L selective inhibition with A-1331852 sensitizes preclinical models of ovarian cancer to belinostat. SKOV3 and IGROV1-R10 cells were treated with both belinostat at cytostatic concentration and either the Bcl-x_L selective inhibitor A-1331852 (Selleckchem) at 1 µM or ABT-737 at 5 µM for 48 hours (A-C). The efficacy of the co-treatments was explored by analyzing the relative number of viable cells using the trypan blue exclusion test (A), the percentage of sub-G1 events obtained by flow cytometry (B) and the cleavage of caspase-3 and PARP detected by western blot (C). The OV-009_T PDTO were treated for 48 hours with belinostat at 0.5 µM (low-toxic concentration) and either A-1331852 at 0.1 µM or ABT-737 at 2 µM. The effect of these combinations was analyzed by evaluating the viability of the PDTO using the CellTiter-Glo® 3D cell viability assay (D). The results shown in the graphs are expressed as the mean ± SD (error bars) of three (A-B) or two (D) independent experiments. **p*<0.05; ***p*<0.01; ****p*<0.001 (Student's t-test)
